# Insights into the efficient degradation mechanism of extracellular proteases mediated by *Purpureocillium lilacinum*

**DOI:** 10.3389/fmicb.2024.1404439

**Published:** 2024-07-08

**Authors:** Xiujun Zhang, Yuhong Yang, Li Liu, Xin Sui, Ramon Santos Bermudez, Lushan Wang, Wenxing He, Huilian Xu

**Affiliations:** ^1^School of Biological Science and Technology, University of Jinan, Jinan, China; ^2^State Key Laboratory of Microbial Technology, Microbial Technology Institute, Shandong University, Qingdao, China

**Keywords:** nematophagous fungi, *Purpureocillium lilacinum*, serine protease, GATA transcription factors, nematodes

## Abstract

Protease secretion is crucial for degrading nematode cuticles using nematophagous fungus *Purpureocillium lilacinum*, but the secretion pattern of protease remains poorly understood. This study aimed to explore the degradation mechanism of proteases by investigating the characteristics of protease secretion under various carbon and nitrogen sources, and different carbon to nitrogen (C:N) ratios in *P. lilacinum*. The results showed that corn flour as a carbon source and yeast extract as a nitrogen source specifically induced protease secretion in *P. lilacinum. P. lilacinum* produced significant amounts of gelatinase and casein enzyme at C:N ratios of 10:1, 20:1, and 40:1, indicating that higher C:N ratios were more beneficial for secreting extracellular proteases. Proteomic analysis revealed 14 proteases, including 4 S8 serine endopeptidases and one M28 aminopeptidase. Among four S8 serine peptidases, Alp1 exhibited a high secretion level at C:N ratio less than 5:1, whereas PR1C, PR1D, and P32 displayed higher secretion levels at higher C:N ratios. In addition, the transcription levels of GATA transcription factors were investigated, revealing that Asd-4, A0A179G170, and A0A179HGL4 were more prevalent at a C:N ratio of 40:1. In contrast, the transcription levels of SREP, AreA, and NsdD were higher at lower C:N ratios. The putative regulatory profile of extracellular protease production in *P. lilacinum*, induced by different C:N ratios, was analyzed. The findings offered insights into the complexity of protease production and aided in the hydrolytic degradation of nematode cuticles.

## 1 Introduction

Plant-parasitic nematodes (PPNs) cause significant yield declines in crop plants, resulting in annual economic losses exceeding $157 billion (Abad et al., 2008). Managing crop diseases caused by nematodes is essential. Currently, agricultural chemical pesticides are the most effective means of controlling PPNs (Pires et al., 2022). However, the adverse effects of chemical pesticides pose significant threats to public health and ecological balance (Burns et al., 2023). Thus, safe and effective strategies are needed to deter and control nematode infestations. The use of natural microbial predators for the biocontrol of nematodes has gained increasing scientific interest, aiming to understand the complex interactions between nematodes and their microbial adversaries (Wang et al., 2022).

Nematophagous fungi, which have the distinctive ability to infect and eliminate nematodes, have emerged as potential biological agents for controlling detrimental nematode populations. Researchers have recently proposed utilizing these fungi due to their natural propensity for infecting and effectively killing nematodes (Poveda et al., 2020). These fungi are primarily represented by the genera *Purpureocillium*, *Hirsutella*, and *Pochonia* (Topalović et al., 2020). *Purpureocillium lilacinum*, previously known as *Paecilomyces lilacinus*, is a promising and commercially viable biological agent. Numerous studies attempted to ascertain the efficacy of *P. lilacinum* as a biocontrol agent against PPNs, especially the economically important root-knot nematode *Meloidogyne incognita* (Anastasiadis et al., 2008; Singh et al., 2013).

*P. lilacinum* is a nematophagous fungus from the *Ascomycota* phylum, naturally found in the soil and rhizospheres of numerous crops (Baron et al., 2020; Barbosa et al., 2022). It can grow in a wide temperature range (8°C–38°C) and tolerate varying pH levels (Khan and Tanaka, 2023). This soil fungus has been extensively tested as a promising biocontrol agent targeting PPNs in agricultural settings. Pot experiments have demonstrated that *P. lilacinum* effectively controls second-stage juveniles, eggs, and egg masses of root-knot nematodes (Anastasiadis et al., 2008). When applied to the soil, *P. lilacinum* has demonstrated efficacy in controlling the growth of *Meloidogyne javanica* and *Globodera pallida*, with control rates exceeding 70% (Hajji et al., 2016). Based on these findings, *P. lilacinum* has been approved and registered as a biocontrol agent for nematode management (Kiewnick et al., 2011).

*P. lilacinum* combats nematodes in several ways, such as parasitizing eggs, establishing colonies within females, and eliciting systemic resistance. It can produce a diverse array of biologically active secondary metabolites, including polyketides and non-ribosomal peptides such as leucinostatins. These metabolites have various beneficial properties, including nematicidal, anti-viral, antitumor, and phytotoxic effects (Mori et al., 1982; Park et al., 2004; Wang et al., 2016). The nematode cuticle, primarily composed of proteins such as collagens, is a delicate and flexible exoskeleton (Cox et al., 1981). *P. lilacinum* produces a wide range of carbohydrate hydrolases and proteolytic enzymes, including serine proteases and chitinases, which aid in breaking down the nematode cuticle and facilitate penetration (Wang et al., 2010).

In recent years, significant progress has been made in purifying and identifying cuticle-degrading proteases in fungi such as *Arthrobotrys oligospora*, *Metacordyceps chlamydosporia* (syn. *Verticillium chlamydosporium*), and *Lecanicillium psalliotae* (Segers et al., 1994; Tunlid et al., 1994; Minglian et al., 2004). In 1990, the first pathogenicity-associated serine protease, named P32, was extracted and characterized from the nematode-endoparasitic fungus *Verticillium suchlasporium* (syn. *Pochonia suchlasporia*). It degrades nematode eggshell proteins (Lopez-Llorca, 1990; Lopez-Llorca and Claugher, 1990). Subsequent studies led to the purification and characterization of three additional serine proteases: PII from the nematode-trapping fungus *A. oligospora*, VCP1 from the nematode-endoparasitic fungus *Pochonia chlamydosporia*, and pSP-3 from *P. lilacinum* (Segers et al., 1994; Tunlid et al., 1994; Bonants et al., 1995). Further investigations have resulted in the discovery of an increasing number of nematophagous fungal serine proteases. These include DS1 from *Dactylella shizishanna* (Wang R. B. et al., 2006), Mlx from *Monacrosporium microscaphoides* (Wang M. et al., 2006), PrC from *Clonostachys rosea* (syn. *Gliocladium roseum*) (Li et al., 2006), and Ver112 from *Lecanicillium psalliotae* (Yang et al., 2005). Despite the known hydrolytic activity of these extracellular serine proteases against the nematode cuticle, the detailed regulatory mechanisms governing their production and activity remain unclear.

Although the understanding of the regulation of protease synthesis and the signal transduction pathways controlling protease expression in fungi is still limited, extracellular protease production is strictly regulated (Gonzalez-Lopez et al., 2002; Snyman et al., 2019). Extracellular protease secretion is typically triggered by various environmental stimuli (McCotter et al., 2016). Protease production and its regulation are influenced by different culture conditions, including variations in carbon and nitrogen sources (McCotter et al., 2016; Sun et al., 2021). Certain preferred nitrogen sources, regulated by nitrogen catabolite repression (NCR), can inhibit the expression of protease genes (Dabas and Morschhauser, 2008). Global regulatory genes that mediate protease production in response to nitrogen sources have been shown to involve GATA transcription factors. These include *areA* in *Aspergillus nidulans*, *Aspergillus oryzae*, and *Trichoderma reesei* (Arst and Cove, 1973; Christensen et al., 1998; Qian et al., 2019), *nit-2* in *Neurospora crassa* (Fu and Marzluf, 1987), *GLN3* and *GAT1* in *Candida albicans* (Dabas and Morschhauser, 2008), AreB in *A. nidulans* (Chudzicka-Ormaniec et al., 2019), and *nre* in *Penicillium chrysogenum* (Haas et al., 1995). The GATA transcription factor AreA is crucial for chromatin remodeling and regulates protease and cellulase expression (Muro-Pastor et al., 1999; Qian et al., 2019). AreB, another GATA-family transcription factor, acts as a negative regulator of nitrogen catabolism in nitrogen-limited environments, potentially affecting fungal growth, asexual development, and conidial germination (Wong et al., 2009). Understanding the secretion patterns and regulatory mechanisms of serine protease from the nematophagous fungus *P. lilacinum* may enhance the knowledge of its hydrolytic activity against nematode cuticles.

This study aimed to enhance the understanding of serine protease regulation in *P. lilacinum*. Various carbon and nitrogen sources, along with different carbon-to-nitrogen (C:N) ratios, were employed to induce the growth and production of extracellular proteases in *P. lilacinum*. The biochemical properties were assessed to characterize the produced proteases. A novel perspective on the intricacy of serine proteases was provided by analyzing the functional degradome profile of *P. lilacinum* using liquid chromatography and tandem mass spectrometry (LC–MS/MS). In addition, qRT-PCR analyses were performed for nine GATA transcription factors in *P. lilacicum* to investigate the potential regulation of different proteases by GATA transcription factors under different C:N ratios.

## 2 Results

### 2.1 Impact of carbon sources on fungal sporulation, growth, and protease secretion of *Purpureocillium lilacinum*

**Figure 1:**
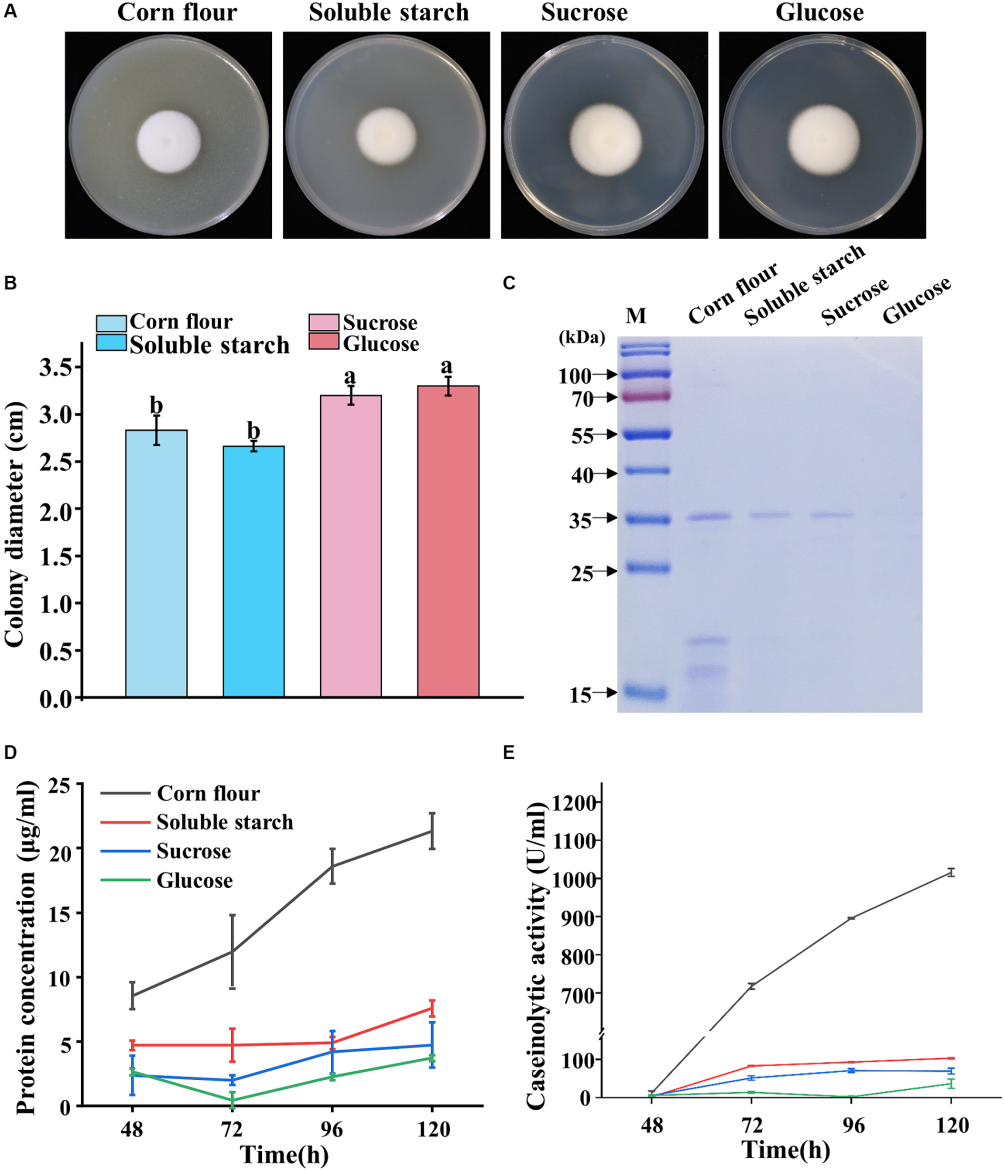
Phenotypic analysis of *P. lilacinum* grown on different carbon sources (corn flour, soluble starch, sucrose, and glucose). **(A)** Colony morphology and **(B)** colony size of 5-day-old cultures on minimal medium supplemented with different carbon sources at 28°C. Exactly 1 μL of a 1 × 10^6^ conidia/mL suspension was spotted onto the center of the various media. Different lowercase letters represent significant differences (*p* < 0.05). **(C)** SDS-PAGE analysis of extracellular proteins from *P. lilacinum* grown for 96 h on different carbon sources. **(D)** Concentration of extracellular protein and **(E)** caseinolytic activity of *P. lilacinum* grown on different carbon sources at different culture times (48, 72, 96, and 120 h). The values represent the mean of three biological replicates, with error bars indicating standard deviations.

Given that the genome of *P. lilacinum* encodes numerous glycoside hydrolases, carbohydrate esterases, and proteases (Varshney et al., 2016; Wang et al., 2016), four carbon sources (corn flour, soluble starch, sucrose, and glucose) were evaluated for their influence on the sporulation and protease secretion of *P. lilacinum*. The mycelial growth and sporulation responded differently to the four carbon sources on solid agar plates. Colonies grown on agar supplemented with corn flour displayed a slight variation, appearing as a white fluffy phenotype, whereas those supplemented with soluble starch, sucrose, and glucose resulted in slightly darker conidia (Figure 1A). *P. lilacinum* exhibited reduced radial growth on medium containing soluble starch compared with that containing sucrose and glucose as carbon sources (corn flour 2.83 ± 0.15 cm, soluble starch 2.67 ± 0.06 cm, sucrose 3.2 ± 0.10 cm, glucose 3.3 ± 0.10 cm) (Figure 1B). The cover slip culture revealed denser mycelium branches under conditions using corn flour or soluble starch as carbon sources, whereas fewer branches were observed in a medium with sucrose and glucose as carbon sources (Supplementary Figure S1A).

Given that some extracellular secreted proteins, including proteases, serve as virulence factors of *P. lilacinum*, destabilizing nematode egg membranes (Wang et al., 2010, 2016), *P. lilacinum* was cultured in a submerged medium using four different carbon sources to examine the extracellular secreted proteins, particularly proteases. Biochemical assays were performed on the filtered culture supernatants, which were analyzed by sodium dodecyl sulfate–polyacrylamide gel electrophoresis (SDS-PAGE) (Figure 1C). Equal volumes of supernatants were loaded for analysis. Almost no protein bands were observed in the culture medium containing glucose as the carbon source, whereas one to several protein bands could be observed in culture media containing corn flour, starch, or sucrose (Figure 1C). Considering that the protein concentration may be influenced by different carbon sources, the protein concentrations were determined. The extracellular protein concentration reached 21.33 μg/mL in the culture medium with corn flour, which was significantly higher than that in the medium with soluble starch, sucrose, or glucose as carbon sources (Figure 1D). Then, the levels of extracellular secreted protease were assayed (Figure 1E). In media using corn flour as the carbon source, the extracellular proteases exhibited the highest level of activity, reaching 1015.45 IU/mL after 5 days of cultivation (Figure 1E), suggesting that corn flour might specifically induce protease secretion in *P. lilacinum*.

### 2.2 Impact of nitrogen sources on fungal sporulation, growth, and protease secretion of *Purpureocillium lilacinum*

**Figure 2:**
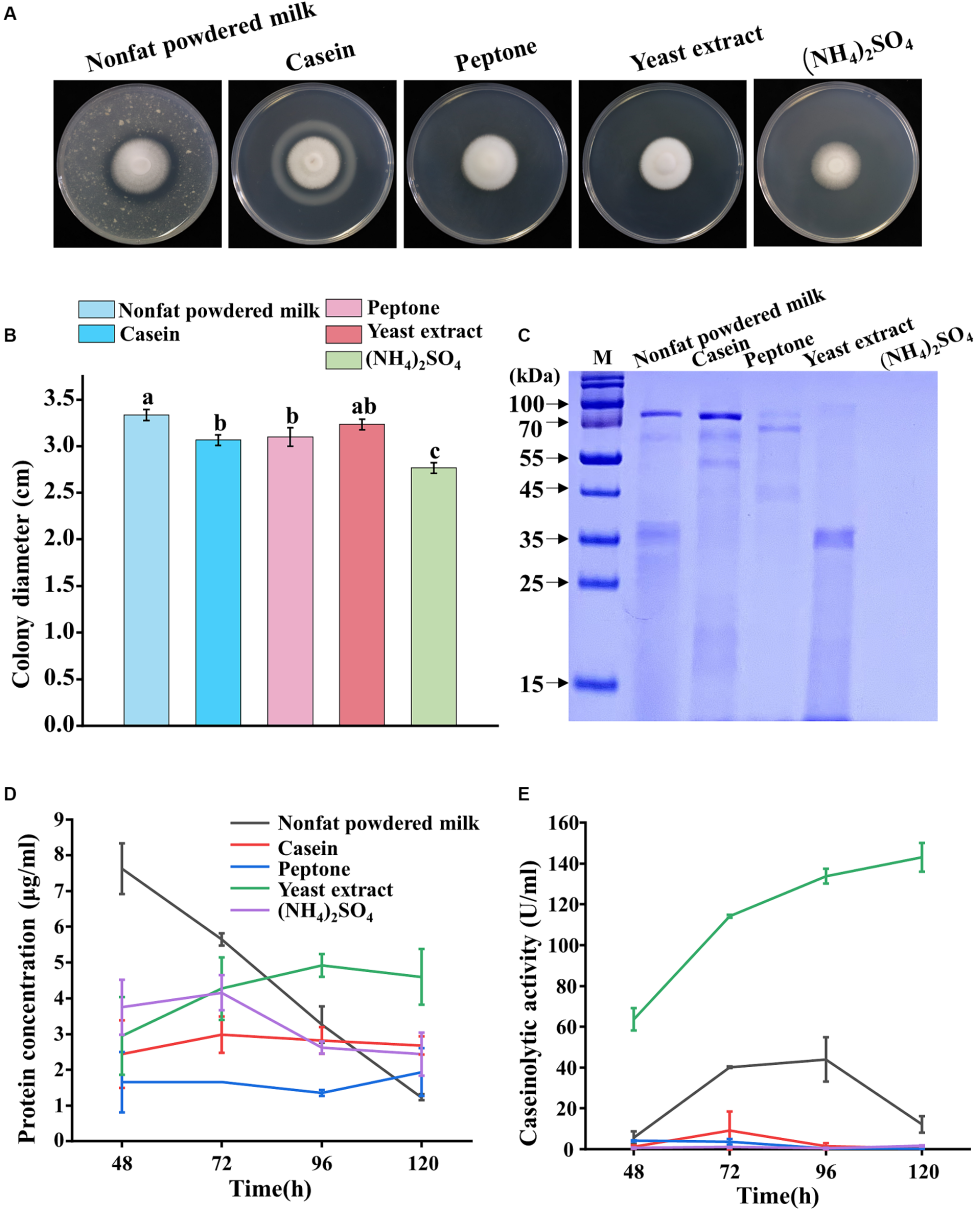
Phenotypic analysis of *P. lilacinum* grown on different nitrogen sources (nonfat powdered milk, casein, peptone, yeast extract, and (NH_4_)_2_SO_4_). **(A)** Colony morphology and **(B)** colony size of 5-day-old cultures on minimal medium supplemented with different nitrogen sources at 28°C. Exactly 1 μL of 1 × 10^6^ conidia/mL suspension was spotted onto the center of the various media. Different lowercase letters represent significant differences (*p* < 0.05). **(C)** SDS-PAGE analysis of extracellular proteins from *P. lilacinum* grown for 96 h on different nitrogen sources. **(D)** Concentration of extracellular protein and **(E)** caseinolytic activity of *P. lilacinum* grown on different nitrogen sources. The values represent the mean of three biological replicates, with error bars indicating standard deviations.

Five nitrogen sources (nonfat powdered milk, casein, peptone, yeast extract, and (NH_4_)_2_SO_4_) were evaluated for their effects on the sporulation growth and protease secretion of *P. lilacinum*. The results revealed that *P. lilacinum* exhibited a white fluffy phenotype, observed on the medium containing yeast extract, whereas slightly darker conidia were produced on cultures supplemented with nonfat powdered milk, casein, peptone, and (NH_4_)_2_SO_4_ (Figure 2A). *P. lilacinum* grew slowly and showed reduced radial growth after 5 days of growth, with notable differences observed in cultures supplemented with (NH_4_)_2_SO_4_ compared with media using nonfat powdered milk, casein, peptone, and yeast extract as nitrogen sources (nonfat powdered milk 3.33 ± 0.06 cm, casein 3.07 ± 0.06 cm, peptone 3.1 ± 0.10 cm, yeast extract 3.23 ± 0.06 cm, and (NH_4_)_2_SO_4_ 2.77 ± 0.06 cm) (Figure 2B). The cover slip culture showed that the mycelium branches were denser under conditions using nonfat powdered milk and yeast extract as nitrogen sources (Supplementary Figure S1B).

Then, *P. lilacinum* was cultivated in a submerged medium with five different nitrogen sources. SDS-PAGE analysis showed clear protein bands when cultured with nonfat powdered milk, casein, peptone, or yeast extract as nitrogen sources, whereas no visible protein bands were observed when (NH_4_)_2_SO_4_ was used (Figure 2C). The extracellular protein concentration reached 4.60 μg/mL in a culture medium with yeast extract after 5 days of cultivation, which was slightly higher than that in the medium with other nitrogen sources (Figure 2D). Consistent with the extracellular protein concentration, the fungus produced significant amounts of extracellular proteases, with a maximum level of 143.02 U/mL in a culture medium with yeast extract after 5 days of incubation (Figure 2E). In contrast, when peptone, casein, and (NH_4_)_2_SO_4_ were used as nitrogen sources, the concentration of proteases was almost undetectable (peptone 0.28 U/mL, casein 0.49 U/mL, and (NH_4_)_2_SO_4_ 1.68 U/mL after 5 days of incubation, respectively) (Figure 2E). The results suggested that yeast extract might specifically induce protease secretion in *P. lilacinum*.

### 2.3 Impact of the C:N ratio on fungal sporulation, growth, and protease secretion of *Purpureocillium lilacinum*

**Figure 3:**
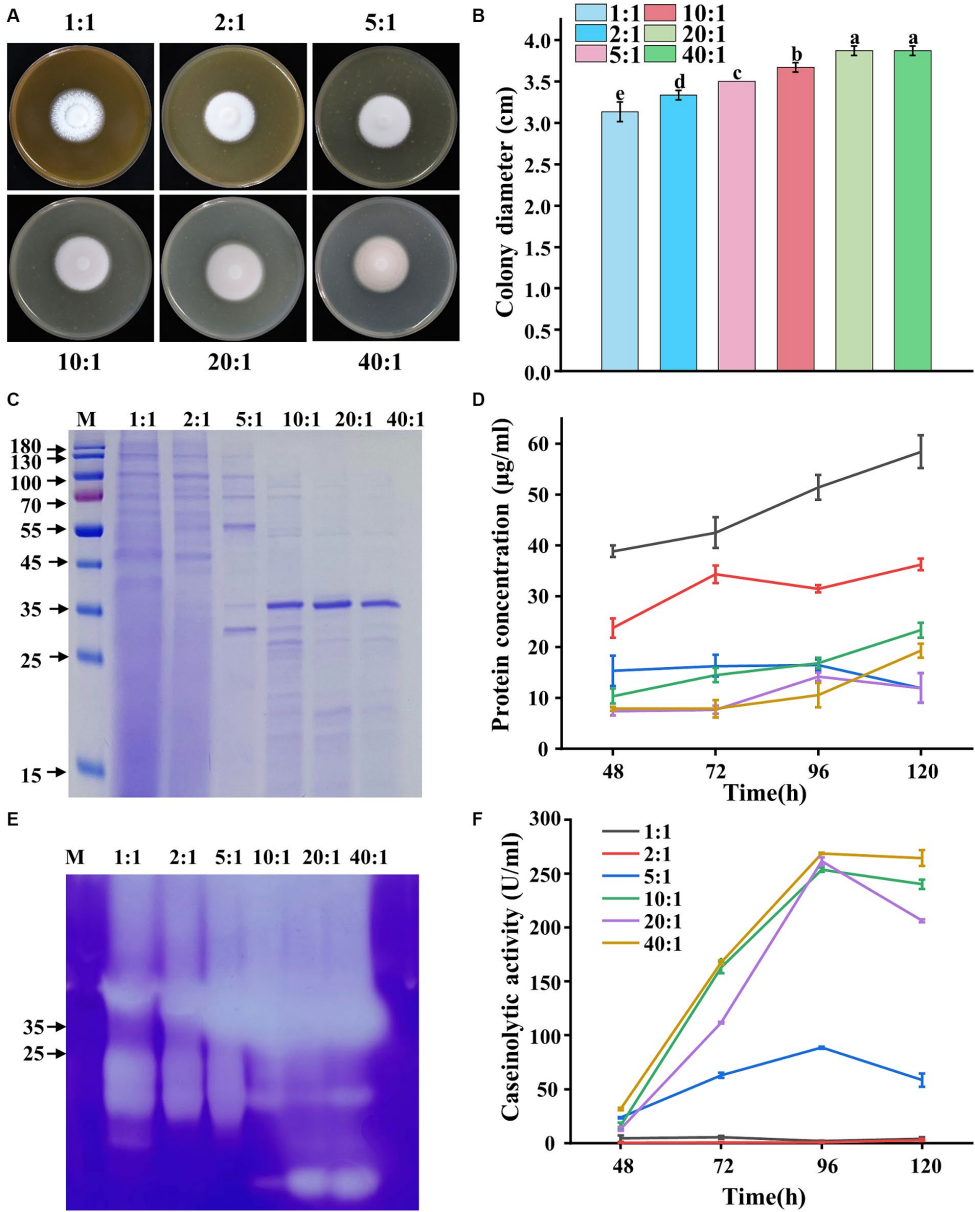
Phenotypic analysis of *P. lilacinum* grown on different C:N ratios (1:1, 2:1, 5:1, 10:1, 20:1, and 40:1). **(A)** Colony morphology and **(B)** colony size of 5-day-old cultures on minimal medium supplemented with different C:N ratios at 28°C. Exactly 1 μL of a 1 × 10^6^ conidia/mL suspension was spotted onto the center of the various media. Different lowercase letters indicate significant differences (*p* < 0.05). **(C)** SDS-PAGE analysis of extracellular proteins from *P. lilacinum* grown for 96 h under different C:N ratios. **(D)** Concentration of extracellular protein from *P. lilacinum*. **(E)** Zymography of gelatinase by secreted proteins of *P. lilacinum* grown under different C:N ratios during 96 h of liquid fermentation. **(F)** Caseinolytic activity of *P. lilacinum* grown under different C:N ratios. The values represent the mean of three biological replicates, with error bars indicating standard deviations.

The protease secretion was much higher when corn flour was used as a carbon source compared with other carbon sources (Figure 1). Similarly, yeast extract used as a nitrogen source resulted in higher protease secretion in *P. lilacinum* compared with other nitrogen sources (Figure 2). Environmental factors, particularly C:N ratios, significantly influence the efficacy of active conidia and the growth of the fungus. Therefore, the biological performance was investigated under different C:N ratios (1:1, 2:1, 5:1, 10:1, 20:1, and 40:1). The results indicated that the colony color tended to become purple-red when the C:N ratios increased, especially to 40:1, where the colony exhibited a deep purple color (Figure 3A). Simultaneously, the changes in colony diameter under different C:N ratios were investigated. The results showed that, consistent with the variation in spore color, the colony diameter increased with the increase in C:N ratios (Figure 3B). When the C:N ratio was 40:1, the colony diameter reached 3.87 ± 0.06 cm. In terms of mycelial growth, an increase was observed in the number of mycelial branches when the C:N ratio was 2:1, 5:1, and 10:1 (Supplementary Figure S1C).

Then, *P. lilacinum* was cultured in a submerged medium with different C:N ratios. SDS-PAGE analysis revealed significantly more protein bands and higher protein concentrations when the C:N ratio was 1:1, 2:1, and 5:1 (Figures 3C,D). However, fewer protein bands and lower protein concentrations were observed when the C:N ratio was 10:1, 20:1, and 40:1, although distinct bands were found around 35–45 kDa, indicative of protease bands (Figures 3C,D), were present. Moreover, the variations in gelatinase production, as indicated by activity assessments, were similarly reflected in gelatinase isozyme zymograms, showing abundant secretion of proteases under different C:N ratios. The intensity of protease bands gradually increased with increasing C:N ratios in samples with the same loading amount. The highest secretion of proteases was observed when the C:N ratios were 10:1, 20:1, and 40:1 (Figure 3E). Consistent with the gelatinase profile, *P. lilacinum* produced significant amounts of casein enzyme, reaching its highest level at 253.49, 261.05, and 268.62 U/mL, respectively, at C:N ratios of 10:1, 20:1, and 40:1, after 96 h of cultivation (Figure 3F). In conclusion, higher C:N ratios were more beneficial to the secretion of extracellular proteases in *P. lilacinum*.

### 2.4 Comparative analysis of the functional secretomes of *Purpureocillium lilacinum* when cultured under different C:N ratios

The secreted proteins of *P. lilacinum* cultured for 96 h under different C:N ratios were analyzed using LC–MS/MS. Among these secreted proteins, 517, 317, 114, 48, 42, and 29 proteins were detected when the C:N ratio was 1:1, 2:1, 5:1, 10:1, 20:1, and 40:1, respectively (see Supplementary material S1 for details). It indicated that the C:N ratio significantly impacted the secreted proteins composition of *P. lilacinum*. A higher C:N ratio led to a reduced total number of secreted proteins, suggesting that excessively high C:N ratios might limit the production of secreted proteins (see Supplementary material S1). The top 40 proteins with the highest relative contents are listed in Table 1. Among these top 40 proteins, A0A179HIQ0 showed the highest relative content, particularly when C:N ratios were higher than 10:1 (Table 1). However, the function of this protein remained unknown. The relative content of A0A179HIQ0 was about 31.90, 33.55, and 56.04% when the C:N ratio was 10:1, 20:1, and 40:1, respectively (Table 1). However, further studies are needed to elucidate its function.

**Table 1:** Comparative analysis of the top 40 proteins secreted by *P. lilacinum* under different C:N ratios.

Accession	Description	MW (kDa)	Signal peptide	Relative content (%)					
				1:1	2:2	5:1	10:1	20:1	40:1
A0A179HIQ0	Uncharacterized protein	53.05	Y	ND	ND	ND	31.90	33.55	56.04
A0A179HXB7	D-3-phosphoglycerate dehydrogenase	42.14	Y	0.14	1.64	3.45	19.47	10.94	15.62
A0A179HAK1	60S acidic ribosomal protein P2	10.94	Y	0.30	0.47	0.99	10.05	6.85	ND
A0A179GBK6	Pyridine nucleotide-disulfide oxidoreductase-like protein	43.80	Y	0.03	0.29	0.93	1.25	2.65	12.56
A0A179HAE5	Adenyl-nucleotide exchange factor sse1	82.23	Y	0.02	0.13	0.19	3.60	9.47	4.00
A0A179HSF2	60S ribosomal protein L7	28.58	Y	ND	ND	ND	12.46	ND	ND
A0A179GN57	β-flanking protein	22.36	Y	ND	0.34	2.56	ND	ND	7.91
A0A179H0N1	Allergen Asp F4-like protein	29.92	Y	2.55	2.20	4.58	0.25	ND	ND
A0A179HHZ0	β-glucosidase	95.08	Y	ND	ND	ND	ND	7.31	1.44
A8QMW6	Proteinase T-like protein P32	40.25	Y	0.02	0.05	0.16	5.45	ND	2.83
A0A179HWZ1	Subtilisin-like serine protease PR1C	92.40	Y	ND	ND	ND	0.24	1.43	6.57
A0A179EYL3	β-glucosidase	72.06	Y	1.64	1.86	3.20	ND	ND	ND
A0A179H6P2	Thioredoxin	11.63	Y	0.32	ND	ND	ND	ND	4.91
A0A179HVP5	Monosaccharide transporter	60.38	Y	0.85	0.64	2.02	0.79	0.62	0.20
A0A179I0I5	4-aminobutyrate aminotransferase	55.39	Y	0.10	0.69	3.27	ND	ND	ND
A0A179HQF6	Glucose-6-phosphate 1-dehydrogenase	58.19	Y	ND	ND	ND	0.93	1.34	1.37
A0A179HJB3	Ig group 2 domain-containingprotein	50.18	Y	2.29	0.02	ND	ND	0.76	ND
A0A179GTL5	α-glucosidase	103.42	Y	0.09	0.17	0.49	0.93	1.01	0.33
A0A179HMA7	Ser-thr-rich glycosyl-phosphatidyl-inositol-anchored membrane family domain-containing protein	25.67	Y	1.36	1.38	ND	ND	0.26	ND
A0A179GSU7	Glutathione S-transferase	24.67	Y	0.07	0.13	0.31	1.78	0.69	ND
A0A179HV48	Peroxiredoxin type-2	17.87	Y	ND	ND	ND	1.44	1.14	ND
A0A179G2L6	Glycoside hydrolase family 64	47.27	Y	1.18	1.08	ND	ND	ND	ND
A0A179HSJ3	Amidase family protein	62.94	Y	0.26	0.49	1.51	ND	ND	ND
A0A179HGN6	90S preribosome component RRP12	138.39	Y	0.64	0.71	0.71	0.02	0.04	0.04
A0A179GM89	RanBP1 domain-containing protein	27.68	Y	0.16	0.26	1.27	0.40	ND	ND
A0A179HN00	Chitinase	63.84	Y	0.91	0.55	0.29	ND	ND	0.15
A0A179H777	Uncharacterized protein	156.75	Y	0.08	0.25	0.28	0.42	0.46	0.33
A0A179GCA7	Endoplasmic reticulum chaperone BiP	72.56	Y	0.16	0.28	1.33	ND	ND	ND
A0A179GQM7	Protein reated to secreted protein-sviceus	44.31	Y	0.38	0.36	0.15	ND	0.65	0.19
A0A179HKU3	Coagulation factor 5/8 type domain-containing protein	39.93	Y	0.44	0.85	0.41	ND	ND	ND
A0A179H497	Stress protein DDR48	29.75	Y	0.24	0.58	0.85	ND	ND	ND
A0A179GT65	Guanine nucleotide-binding protein β subunit-like protein	35.07	Y	0.30	0.46	0.68	ND	ND	ND
A0A179GX87	Alkaline serine protease Alp1	41.83	Y	0.51	0.82	ND	ND	ND	ND
A0A179H6B2	Formate dehydrogenase	47.07	Y	0.38	0.13	0.81	ND	ND	ND
A0A179GSG4	Glucan 1,3-β-glucosidase	42.63	Y	0.49	0.30	0.14	ND	0.35	ND
A0A179HRK6	Phenazine biosynthesis PhzC/PhzF protein	36.56	Y	0.07	0.31	0.88	ND	ND	ND
A0A179HDV3	Peptidase inhibitor i9 domain-containing protein	10.67	Y	0.36	0.19	0.64	ND	ND	ND
A0A179GM39	NADP-dependent glycerol dehydrogenase	36.46	Y	0.11	0.60	0.48	ND	ND	ND
A0A179HH73	Cerevisin	57.69	Y	0.03	0.40	0.72	ND	ND	ND
A0A179HWP5	Cell wall protein	27.05	Y	0.62	0.48	ND	ND	ND	ND

ND, not detectable.

In addition, among the top 40 proteins, 6 glycoside hydrolases (GHs) were identified, including 3 β-glucosidase (A0A179HHZ0, A0A179EYL3, and A0A179GSG4), 1 α-glucosidase (A0A179GTL5), 1 glycoside hydrolase family 64 (A0A179G2L6), and 1 chitinase (A0A179HN00) (highlighted in green in Table 1). Furthermore, three proteases were detected in the secretome, including A8QMW6, A0A179HWZ1, and A0A179GX87, encoding the proteinase T-like protein P32, subtilisin-like serine protease PR1C, and alkaline serine protease Alp1, respectively (highlighted in orange in Table 1). These findings were as expected, as GHs and protease are known virulence factors of *P. lilacinum*, contributing to the destabilization of nematode egg membranes (Wang et al., 2010, 2016).

**Figure 4:**
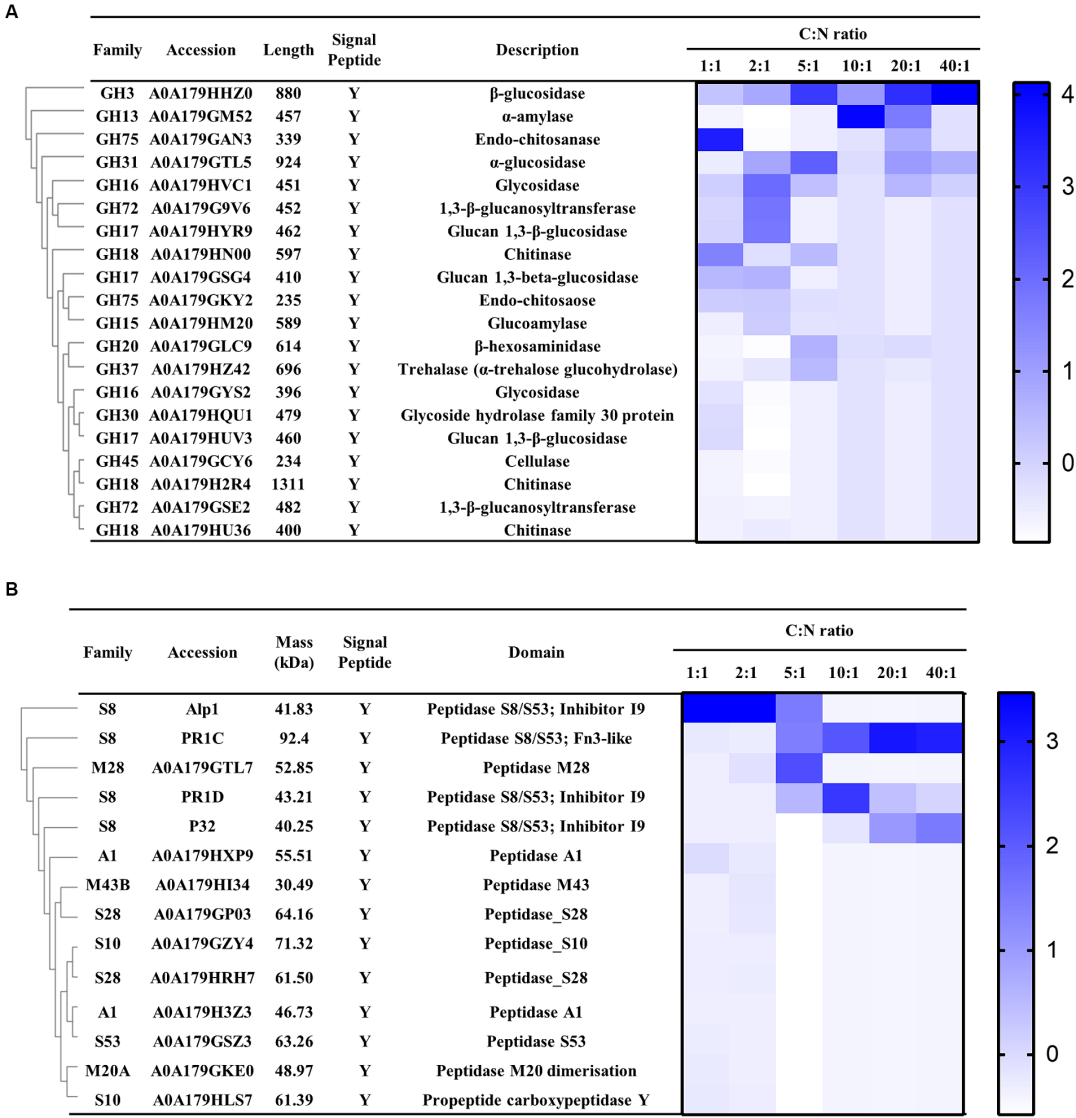
Expression profile of *P. lilacinum* grown for 96 h under different C:N ratios (1:1, 2:1, 5:1, 10:1, 20:1, and 40:1), detected by LC–MS/MS. **(A)** Cluster analysis of extracellular glycoside hydrolases secreted by *P. lilacinum*. **(B)** Cluster analysis of extracellular proteases. The color intensity corresponds to the relative content of each enzyme.

In the genome of *P. lilacinum*, 253 genes encoding GHs were identified, belonging to 55 families (Wang et al., 2016). Among these, 85 glycoside hydrolase–encoding genes contained a putative signal peptide coding sequence, crucial for infecting nematode eggs. In the secreted proteins of *P. lilacinum*, 20 glycoside hydrolases were detected, including β-glucosidase (GH3), α-amylase (GH13), endo-chitosanase (GH75), α-glucosidase (GH31), glycosidase (GH16), and 1,3-β-glucanosyltransferase (GH72) (Figure 4A). Additionally, three chitinases (GH18), including A0A179HN00, A0A179H2R4, and A0A179HU36, were identified (Figure 4A), which degraded the chitin found in the chitin protein complex of the nematode eggshell (Bird and McClure, 2009). In the genome of *P. lilacinum*, 179 genes encoded proteases, with 70 protease-encoding genes containing a putative signal peptide coding sequence crucial for infecting nematode eggs. In the secretome, 14 extracellular proteases were detected, mainly including 4 proteases belonging to the serine endopeptidase family S8: Alp1, PR1C, P32, and PR1D (A0A179GPC6), along with one M28 aminopeptidase (A0A179GTL7) (Figure 4B). Among the serine peptidases of the S8 family, Alp1 had high secretion levels when the C:N ratio was less than 5:1 (C:N = 1:1, 2:1, and 5:1), whereas PR1C, PR1D, and P32 had higher secretion levels at higher C:N ratios (C:N = 10:1, 20:1, and 40:1) (Figure 4B). The substantial secretion of PR1C, PR1D, and P32 at higher C:N ratios significantly contributed to their casein enzymatic activity, which was significantly higher compared with that at lower C:N ratios (Figure 3F). These findings revealed that Alp1 might contribute to the degradation of the nematode cuticle at lower C:N ratios, whereas PR1C, PR1D, and P32 might play crucial roles in the degradation of the nematode cuticle at higher C:N ratios.

### 2.5 Quantitative transcript analysis of key GATA transcription factors under different C:N ratios

**Figure 5:**
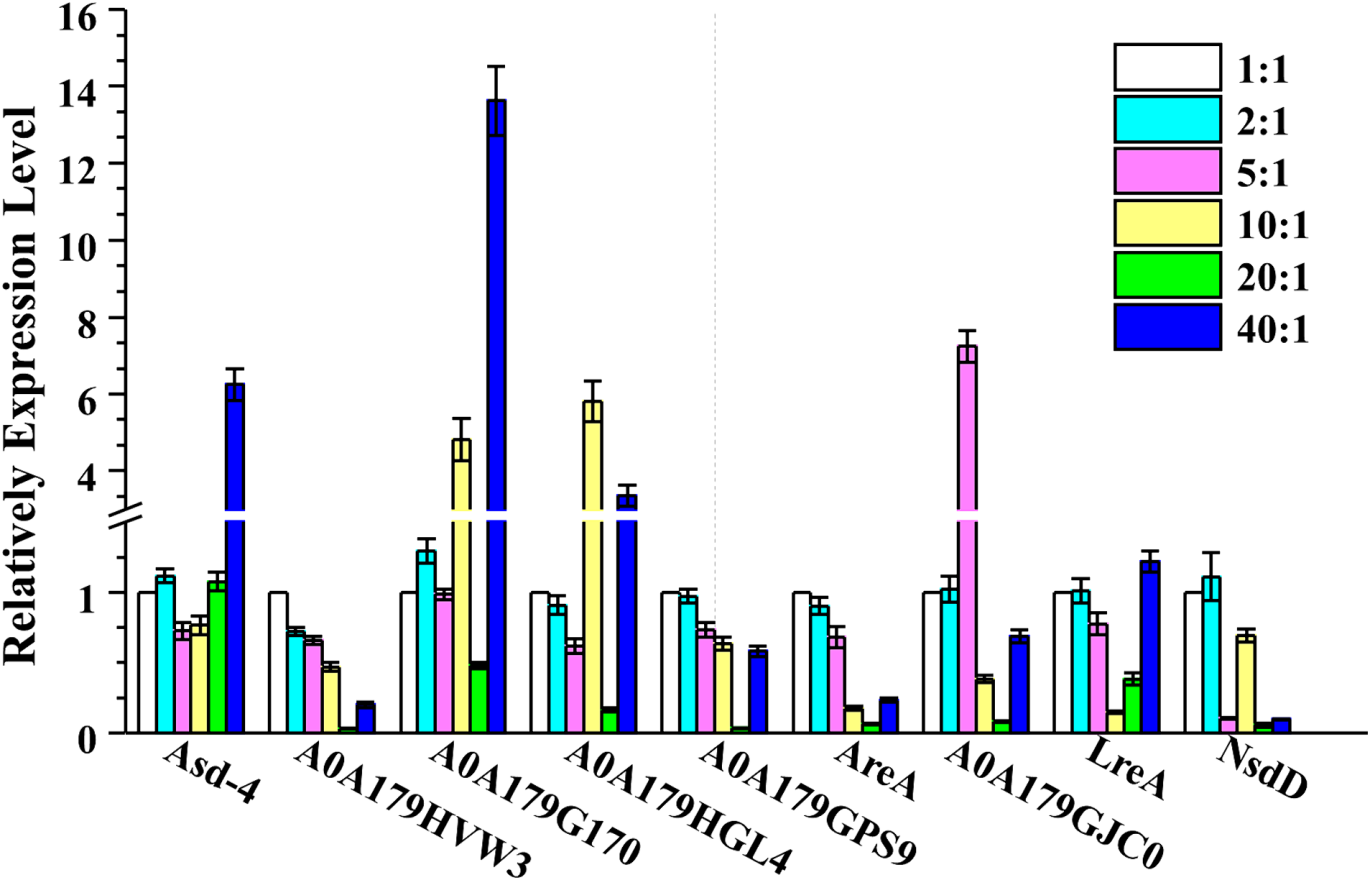
Transcription levels of nine GATA transcription factors, including Asd-4, AreA, LreA, NsdD, SREP, A0A179G170, A0A179HGL4, A0A179GPS9, and A0A179GJC0, were determined by qRT-PCR after cultivating *P. lilacinum* for 24 h under different C:N ratios (1:1, 2:1, 5:1, 10:1, 20:1, and 40:1). The *Actin* gene was used for data normalization. Values represent the mean of three biological replicates, with error bars indicating standard deviations.

In general, *P. lilacinum* secretes many extracellular proteases during its growth, especially under varying C:N ratios, leading to the secretion of different proteases. GATA transcription factors, such as Are1, recognized as an orthologue of the *Aspergillus* global nitrogen regulator AREA, have been characterized for their role in regulating protease production in fungi, including *T. reesei* (Qian et al., 2019). The qRT-PCR analyses (at 24 h) for nine GATA transcription factors in *P. lilacicum* were conducted to investigate the potential regulation of different proteases by GATA transcription factors under different C:N ratios. These included Asd-4 (A0A179I058), AreA (A0A179H9V6), LreA (A0A179HUZ3), NsdD (A0A179GBX3), SREP (A0A179HVW3), A0A179G170, A0A179HGL4, A0A179GPS9, and A0A179GJC0, containing one or more highly conserved GATA zinc finger motifs recognizing consensus 5’-HGATAR-3’ DNA sequences (Scazzocchio, 2000). The transcription levels of Asd-4, A0A179G170, and A0A179HGL4 in *P. lilacinum* increased by fold-changes of 6.26, 13.62, and 3.36, respectively, at a C:N ratio of 40:1 compared with a ratio of 1:1. Conversely, under the same conditions, the transcription levels of SREP, AreA, and NsdD at a C:N ratio of 40:1 were 0.20, 0.23, and 0.09 times lower than those at a ratio of 1:1, respectively (Figure 5).

**Figure 6:**
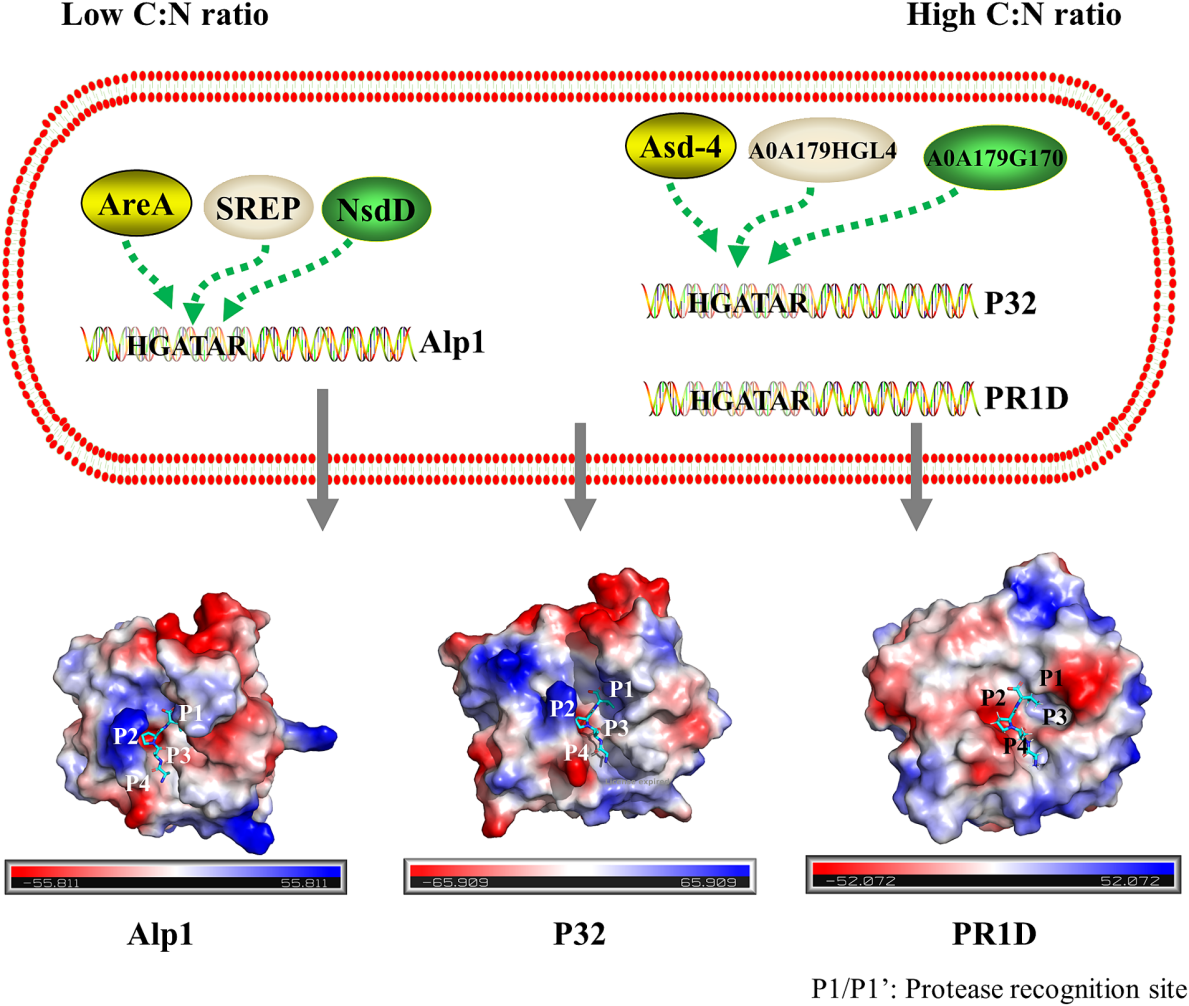
Schematic description of the regulation of the main secreted S8 family serine proteases by GATA transcription factors in *P. lilacinum*. Under a low C:N ratio, the alkaline serine protease Alp1 was regulated by GATA transcription factors SREP, AreA, and NsdD. Under a high C:N ratio, the proteinase T-like protein P32 and serine endopeptidases PR1D were regulated by GATA transcription factors Asd-4, A0A179G170, and A0A179HGL4. The electrostatic potential distribution on the surface of Alp1, P32, and PR1D is shown, with the electrostatic potential colored from red (negative) to blue (positive). The binding sites on the peptide substrate interacting with the protease-binding pockets S4–S4’ were labeled as P4, P3, P2, P1, P1’, P2’, P3’, and P4’, from N-terminal (N) to C-terminal (C). Substrate cleavage occurred between P1 and P1’.

In many fungi, the expression of the protease genes is regulated by GATA transcription factors (Kudla et al., 1990; Marzluf, 1997; Ward et al., 2012). GATA motifs are present in the upstream regions of the key S8 serine endopeptidases PR1D, Alp1, and P32 (Supplementary Figure S2), suggesting that these three proteases were likely regulated by GATA transcription factors. The findings of this study showed that Alp1 exhibited high secretion levels when the C:N ratio was 1:1, whereas PR1D and P32 displayed higher secretion levels at a C:N ratio of 40:1. This suggested that the expression of S8 serine endopeptidases PR1D and P32 was regulated by the GATA transcription factors Asd-4, A0A179G170, and A0A179HGL4. In contrast, the expression of the S8 serine endopeptidases Alp1 was regulated by the GATA transcription factors SREP, AreA, and NsdD (Figure 6). However, further studies are needed to confirm these regulatory mechanisms.

## 3 Discussion

The release of protease enzymes by nematophagous fungi plays a crucial role in degrading the nematode cuticle and facilitating the penetration process. *P. lilacinum* is notably adept at producing a wide range of extracellular proteases. However, the detailed profiles of these extracellular proteases in *P. lilacinum* remain scarce. Previous studies revealed that environmental factors, such as carbon and nitrogen sources and the C:N ratio, significantly impacted fungal protease production. This investigation sought to understand how different carbon and nitrogen sources, and varying C:N ratios, influenced the growth of fungi and the secretion of proteases. Protease classifications under various C:N ratios were examined through LC–MS/MS analysis.

The results showed distinct levels of protease secretion in *P. lilacinum* when exposed to various carbon and nitrogen sources. Notably, complex carbon sources such as corn flour significantly enhanced protease production, in contrast to minimal production with pure carbon sources such as glucose and sucrose. Zymography indicated the most potent proteolytic activity with complex carbon sources, particularly corn flour. These observations aligned with prior findings that complex substrates, such as corn flour and gluten meal, significantly stimulated the gene expression of *Aspergillus niger* AS3.350 protease (*pepD*), and exhibited higher activity (Ke et al., 2019). Conversely, some pure sugars had little to no effect due to carbon catabolite repression (Katz et al., 2000; Akhavan Sepahy and Jabalameli, 2011). Furthermore, the transcription factor CreA repressed the production of proteases in *A. nidulans* when the preferred carbon source glucose was present (Katz et al., 2008). These findings indicated that carbon sources modulated protease production, and the absence of a preferred carbon source induced protease expression.

Yeast extract significantly enhanced protease secretion in *P. lilacinum*, whereas preferred nitrogen sources such as (NH_4_)_2_SO_4_ significantly reduced it. This suggested that preferred nitrogen sources suppressed protease production through NCR, mediated by GATA transcription factors (Dabas and Morschhauser, 2008; Katz et al., 2008). Fungi have evolved complex genetic regulatory systems, heavily reliant on transcription factors, enabling them to produce appropriate enzymes in response to environmental changes in carbon and nitrogen sources (Fernandez et al., 2012). A wide range of transcription factors associated with the regulation of secreted enzyme production has been identified in fungi (Ilmen et al., 1996; van Peij et al., 1998; Saloheimo et al., 2000). For example, the expression of extracellular protease genes in *T. reesei* was modulated by the GATA transcription factor AreA (Qian et al., 2019). The upstream regions of key S8 serine endopeptidases PR1D, Alp1, and P32 (Supplementary Figure S2) contain GATA motifs, indicating that these proteases may be regulated by GATA transcription factors. AreA orthologues, which are transcription activators in some filamentous fungi, are known to activate the production of extracellular proteases. It was, therefore, not surprising that AreA exhibited high transcription levels at lower C:N ratios. These findings indicated that the regulation of protease production in *P. lilacinum* was influenced by the availability of preferred nitrogen sources. A similar nitrogen-controlled regulatory mechanism may exist for protease production in *P. lilacinum* as in other filamentous fungi.

Studies have shown that efficiently degrading complex substrate proteins often requires the collaborative action of multiple enzymes, as a single protease may not be sufficient for this task (Yamamura et al., 2002). *P. lilacinum* predominantly secretes serine proteases, specifically subtilisins (S8) and serine carboxyproteases (S10), which are crucial for nematode infection (Yang et al., 2007; Wang et al., 2016). LC–MS/MS analyses identified 14 proteases in *P. lilacinum*, primarily consisting of four S8 serine endopeptidases: Alp1, PR1C, PR1D, and P32. PR1C is a subtilisin-like Pr1 protease. Despite its molecular weight of 92.4 kDa, which differs from the 35–45 kDa molecular weight range typically observed in virulence-related serine proteases of most nematophagous fungi (Lopez-Llorca and Claugher, 1990; Burns et al., 2023), the homologous proteins of PR1C have been reported as significant virulent factors due to their activity against insect cuticles, for instance, such as in *Beauveria bassiana* (Sheng et al., 2006) and *Metarhizium anisopliae* (St Leger et al., 1992). The phylogenetic analysis suggested that these proteases diverged into two groups with distinct homologies and conserved domains, emphasizing their distinct roles and nonredundant functions at various C:N ratios. One subclade included three S8 serine endopeptidases (Alp1, PR1D, and P32), whereas another subclade contained one S8 serine endopeptidase (PR1C) (Supplementary Figure S2B). PR1D showed high homology with serine proteases Alp1 and P32 (45.06 and 32.70% identity, respectively). All three serine proteases shared the same conserved domain (inhibitor I9 and Peptidase_S8/S53 domain), whereas PR1C contained the conserved Peptidase_S8_5 domain and Peptidase_S8/S53 domain according to the Pfam analysis (Finn et al., 2016) (Supplementary Figure S2B). PR1C and PR1D are subtilisin-like Pr1 proteases, which are considered key virulence factors (St Leger et al., 1996; St Leger and Wang, 2010). PR1C was mainly secreted at higher C:N ratios, whereas PR1D was primarily secreted at a ratio of 10:1 (Figure 4B), suggesting that their functions may be nonredundant (Bagga et al., 2004).

Although S8 serine endopeptidases operate via a catalytic triad mechanism, they exhibit broad substrate specificity, often preferring to cleave after large hydrophobic amino acid residues (Lizbeth, 2003; Laskar et al., 2012). The substrate specificity of each S8 serine endopeptidase is primarily determined by the unique architecture of its active site (Klein et al., 2017). This results in variations in the types of S8 serine endopeptidases secreted under different C:N ratios. As shown in Figure 6 and Supplementary Figure S3, the active site architecture of PR1D, Alp1, and P32 differed from that of PR1C, suggesting that PR1C had a specific preference for substrates. At high C:N ratios, a higher quantity of corn flour was observed, which, according to amino acid composition analysis, exhibited a relatively high glycine content (Supplementary Table S1). Glycine was uncharged, aligning with the neutral surface potential of the substrate-binding pocket of PR1C (Supplementary Figure S3). In addition, in nematodes cuticle, collagens constitute around 80% of total protein content (Kingston, 1991). The cuticle collagens exhibit distinctive Gly-X-Y repetitive motifs, where proline and hydroxyproline frequently occupy the positions of X and Y, respectively (Page and Johnstone, 2007). Additionally, these collagens exhibit conserved patterns of cysteine residues (Johnstone, 2000). Glycine and cysteine were uncharged, aligning with the neutral surface potential of the substrate-binding pocket of PR1C (Supplementary Figure S3). It is speculated that PR1C may have a higher preference for degrading nematode cuticles. However, further verification is necessary.

## 4 Materials and methods

### 4.1 Fungal strain and culture conditions

*Purpureocillium lilacinum* PLFJ-1 was sourced from the China General Microbiological Culture Collection Center (CGMCC No. 3.17493) and preserved on potato dextrose agar (PDA) slants at 4°C. For conidial production, the fungus was grown on a PDA medium at 28°C for 5 days. The fungal strains were routinely cultivated on a minimal medium consisting of 0.1% KH_2_PO_4_, 1% NaCl, and 0.01% MgSO_4_, supplemented with different carbon sources, nitrogen sources, and C:N ratios. In the carbon source experiments, the minimal medium was individually supplemented with 20.0 g/L of corn flour, soluble starch, sucrose, or glucose, along with 5.0 g/L of peptone as the organic nitrogen source. For the nitrogen source experiments, the medium was supplemented with 20.0 g/L sucrose and 5.0 g/L of either nonfat powdered milk, casein, peptone, yeast extract, or (NH_4_)_2_SO_4_. Using an elemental analyzer based on dry combustion (Vario EL Cube, Elementar, Germany), analysis of C and N content revealed that the C content of corn flour is 46.7%, and the N content of yeast extract is 10.9%. When investigating the effects of different C:N ratios, the medium was supplemented with 20.0 g/L corn flour, and the initial C:N ratios were adjusted by varying the amount of yeast extract added. The yeast extract concentrations were 169.80, 84.90, 33.96, 16.98, 8.50, and 4.24 g/L, corresponding to C:N ratios of 1:1, 2:1, 5:1, 10:1, 20:1, and 40:1, respectively. These setups allowed for an accurate assessment of how different carbon and nitrogen sources, as well as varying C:N ratios, influenced the growth and protease production of *P. lilacinum*.

### 4.2 Phenotypic analyses

Phenotypic analyses were performed using the minimal medium (0.1% KH_2_PO_4_, 1% NaCl, and 0.01% MgSO_4_) supplemented with different carbon sources, nitrogen sources, and C:N ratios as described earlier. A 1-μL suspension of 1 × 10^6^ conidia/mL was spotted onto the center of the various media and cultured at 28°C for 5 days to assess colony size. Each treatment was replicated three times. For conidiophore observation, 100 μL of conidial suspension with a concentration of 1 × 10^8^ conidia/mL was applied to the medium. Several 18 mm sterile cover slips were placed into the medium at a 45° angle to the agar surface. The plate was incubated at 30°C for 3 days. The nature and morphology of conidiophores were observed, and digital images were captured using an MSHOT-MSX1 microscope equipped with a 40× objective and MSHOT Image Analysis System 1.1.5 software, all from MSHOT, China. Each treatment was replicated three times.

### 4.3 Determination of protease activity and extracellular protein content

The strain was cultured in a PDA medium at 28°C for 18 h. Then, 1 mL of mycelia suspension was harvested for each sample and transferred into 50 mL of inducible medium containing different carbon and nitrogen sources as well as C:N ratios. Fermented broths were collected at various culture times (48, 72, 96, and 120 h), followed by centrifugation at 10,000 *g* at 4°C for 10 min. The obtained crude enzyme was used for subsequent experimental analysis. Three replications were conducted for each treatment.

The Folin-phenol method was used to evaluate caseinolytic activity by applying casein as the substrate, following a protocol delineated by Salim et al. (2017) with minor adjustments. The supernatant, diluted with 50 mM Tris–HCl (pH 8.0), was enriched with 100 μL of diluted enzyme solution and added to a 2% (*w*/*v*) casein substrate. This mixture was then incubated at 40°C for 10 min. The reaction was terminated by adding 200 μL of 0.4 M trichloroacetic acid. After centrifugation at 10,000 *g* at 4°C for 10 min, 100 μL of the supernatant was mixed with 500 μL of 0.4 M Na_2_CO_3_ and 100 μL of Folin-phenol reagent. Then, the mixture was incubated at 40°C for 20 min, followed by the absorbance measurement at 660 nm. The enzymatic activity unit was defined as the quantity of enzyme required to catalyze the hydrolysis of casein, resulting in the production of 1 μmol of tyrosine per minute under uniform temperature conditions.

The protein concentration in the supernatant was determined following the Bradford method (Bradford, 1976), utilizing Coomassie Brilliant Blue G250 dye and bovine serum albumin (0.1 mg/mL) as the calibration standard. Subsequently, SDS-PAGE analysis was conducted. The separation gel was prepared with a 12% (*w*/*v*) concentration, whereas the stacking gel was formulated at 5% (*w*/*v*). Protein samples, subjected to heat treatment at 105°C for 10 min, were loaded onto the gel in 50-μL aliquots. For electrophoresis, a constant voltage is consistently applied across the gel. At the onset of the procedure, set the initial voltage to 80 V. Subsequently, once the indicator dye migrates into the separation gel, adjust the voltage to 120 V and maintain this level until electrophoresis is completed, which typically takes one hour. The protein bands were visualized by staining with Coomassie Brilliant Blue for 30 min and subsequently destained using a solution containing 10% absolute ethanol and 10% glacial acetic acid post-separation.

### 4.4 Zymogram analysis of the extracellular functional degradome

The strains were cultivated following the procedure outlined in the section “Determination of protease activity and extracellular protein content.” After 96 h, the fermented broths were collected and centrifuged to remove the fungal mycelia. Gelatin zymography was used to assess the changes in extracellular functional degradome dynamics. This analytical approach involved using a gel comprising 13.6% (*w*/*v*) SDS-PAGE co-polymerized with a 0.1% (*w*/*v*) gelatin substrate to detect proteolytic activity. The samples were prepared by mixing them with the sample loading buffer at a 4:1 ratio and then directly loaded onto the gel without prior heating. Electrophoresis was conducted under chilled conditions on the ice. Following electrophoresis, SDS was removed from the gel through a series of sequential washes: two 20-min washes with 2.5% (*v*/*v*) Triton X-100, followed by two additional 20-min washes with a mixture of Triton X-100 and 50 mM Tris–HCl at pH 8.0, and finally, two 20-min washes with 50 mM Tris–HCl at pH 8.0 alone. The proteolytic reaction was then initiated in the same buffer and carried out at 37°C for 45 min. Then, the gel was stained with a 0.1% Coomassie Blue R-250 solution to visualize and assess gelatinase activity. The quantitative assessment of gelatin substrate degradation against the stained gelatin background facilitated the determination of degradome component activity.

### 4.5 Analysis of active degradome components using LC–MS/MS

The strains were cultivated as described in the section “Determination of protease activity and extracellular protein content.” After 96 h of fermentation, the fermented broths were collected and centrifuged to remove fungal mycelia. The resulting supernatant was concentrated via ultrafiltration with a molecular weight cutoff of 10 kDa. LC–MS/MS analysis was conducted using the method described in a previous study (Zhang et al., 2015). Protein precipitation from the supernatant was induced using cold acetone in a volume four times that of the supernatant. The resulting precipitate was dissolved in 8 M urea supplemented with 100 mM Tris-Cl at pH 8.5. Protein concentration was determined using the bicinchoninic acid assay. Subsequently, protein modification steps included reduction with Tris(2-carboxyethyl)phosphine (TCEP) and alkylation using iodoacetamide (IA), both performed at 37°C for 1 h. The urea concentration was then diluted to below 2 M with 100 mM Tris–HCl (pH 8.5) while preparing for enzymatic digestion. Proteolytic digestion using trypsin proceeded overnight at a ratio of 1:50 (enzyme:protein, by weight) at 37°C. The digestion was stopped the following day by adjusting the pH to 6.0 using trifluoroacetic acid (TFA). After centrifugation at 12,000 *g* for 15 min, a custom-designed desalting column was employed for peptide purification. The purified peptides were then vacuum-concentrated and stored at −20°C for subsequent analysis.

The Q Exactive HF mass spectrometer, interfaced with the UltiMate 3,000 RSLCnano system, was used for LC–MS/MS data acquisition. Peptide separation was achieved on a C18 analytical column using a binary gradient system of mobile phase A (0.1% formic acid) and phase B (80% acetonitrile with 0.1% formic acid), with a flow rate of 300 nL/min. The data-dependent acquisition mode encompassed a full scan (resolution of 60 K, Automatic Gain Control (AGC) target of 3e6, maximum injection time of 25 ms, scanning mass range of 350–1,500 *m*/*z*), followed by 20 MS/MS scans (resolution of 15 K, AGC target of 1e5, maximum injection time of 50 ms). The higher-energy collisional dissociation was set to 27 energy units, with a precursor ion isolation window of 1.4 Da and a dynamic exclusion time of 24 s to avoid redundant selection of target ions.

### 4.6 Database search

The mass spectrometry data were analyzed using MaxQuant software (version 1.6.6) with the Andromeda search engine. Searches were performed against the *P._lilacinum* proteome reference provided by UniProt. Variable modifications included oxidation (M) and acetylation (protein N-term), whereas carbamidomethylation (C) was a constant modification. Trypsin/P enzyme specificity was used for proteolytic cleavage. Precursor ion tolerance was set to 20 parts per million (ppm) for the initial search and refined to 4.5 ppm for the final analysis. Secondary mass spectrometry match tolerance also adhered to the 20-ppm threshold. The filtering criteria employed a stringent 1% false discovery rate at both protein and peptide levels to ensure reliability. The exclusion criteria eliminated entries from the reversed database, contaminants, and proteins identified by a single modified peptide. The refined dataset obtained was then subjected to subsequent analysis.

### 4.7 RNA extraction and qRT-PCR

The strains were cultivated using the methods outlined in the section titled “Determination of protease activity and extracellular protein content.” The fermented broths were harvested after 18 h and centrifuged to remove the fungal mycelia, which were then pulverized into a powder using liquid nitrogen. Total RNA extraction was performed after 24 h of cultivation using 1 mL of RNAiso reagent (Vazyme, China). Then, the RNA was transcribed into cDNA using the PrimeScript RT Reagent Kit (Vazyme). The expression levels of 9 GATA transcription factors were measured with qRT-PCR and determined by the 2^-ΔΔCt^ methods. The data were normalized with the *act* gene (A0A179GJ26) as a reference gene. The primer pairs used in this study are listed in Supplementary Table S2. All qPCR analyses were performed using SYBR Premix Ex Taq (Vazyme) and a Bio-Rad CFX Connect instrument (Bio-Rad, United States). Each sample was analyzed in three biological replicates to ensure the robustness and reliability of the results.

### 4.8 Statistical analysis

Statistical analysis was conducted to determine the significance of differences between samples using the one-tailed homoscedastic *t* test in Microsoft Office 2016 Excel software, with *p* values calculated to assess significance.

### 4.9 Bioinformatics analysis

The amino acid sequences for structural studies were retrieved from UniProt.^1^ Conserved domain searches were conducted using the Pfam database.^2^ Phylogenetic relationships were inferred by aligning multiple sequences using MEGA 7.0 software, with trees constructed employing both maximum likelihood and neighbor-joining methods as described by Kumar et al. (2016). Protease structures were modeled using AlphaFold 2 (Varadi et al., 2022) and visualized with PyMOL.^3^ Molecular docking for protease–substrate complexes utilized structures of cuticle-degrading protease PL646 (PDB: 3F7O), a proteinase K-like enzyme (PDB: 2B6N), and glutaminyl cyclase (PDB: 6YI1). Putative GATA transcription factors were identified through the MycoCosm database.

## Data availability statement

The original contributions presented in the study are included in the article/Supplementary material further inquiries can be directed to the corresponding authors.

## Author contributions

XZ: Formal analysis, Methodology, Writing – original draft, Writing – review & editing. YY: Formal analysis, Writing – original draft. LL: Formal analysis, Methodology, Writing – original draft. XS: Formal analysis, Methodology, Writing – review & editing. RB: Writing – review & editing. LW: Conceptualization, Writing – review & editing. WH: Conceptualization, Project administration, Writing – review & editing. HX: Project administration, Writing – review & editing.

## Footnotes

^1^https://www.uniprot.org/

^2^http://pfam.xfam.org/

^3^http://www.pymol.org
