# Supplementary material for: Insights into the efficient degradation mechanism of extracellular proteases mediated by Purpureocillium lilacinum
Source: Front Microbiol. 2024 Jul 8;15:1404439. doi: 10.3389/fmicb.2024.1404439 (PMC11260826; doi:10.3389/fmicb.2024.1404439)
Supplement: Supplementary file 1 [file Table_1.DOCX]

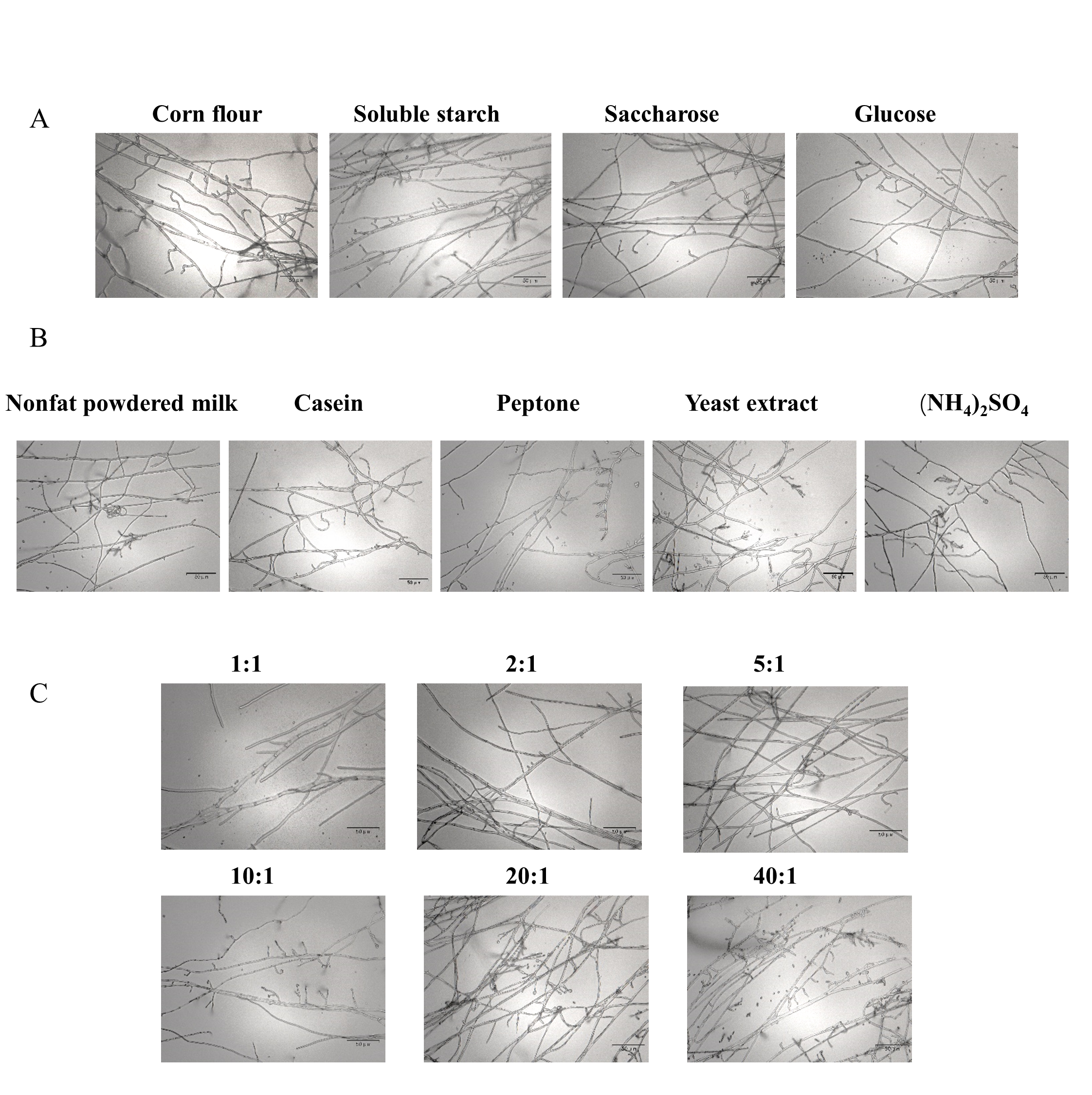
Supplementary Material

Supplementary Figure 1. Observation of conidiophore and mycelia of P. lilacinum under different (A) carbon and (B) nitrogen sources, as well as the (C) carbon to nitrogen ratio (C:N).


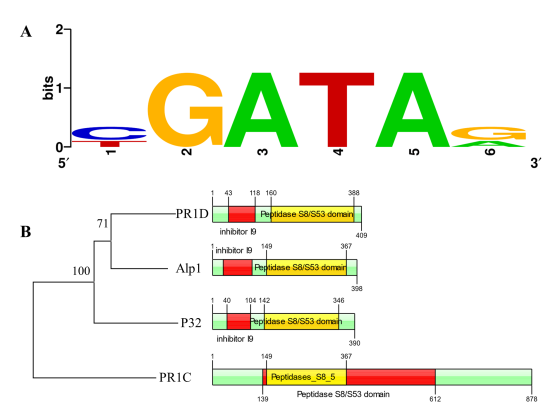


**Supplementary Figure 2.** Analysis of the four S8 serine endopeptidases. (A) GATA motifs in the upstream region of the four S8 serine endopeptidases. (B) Phylogenetic analysis of four S8 serine endopeptidases PR1D, Alp1, P32 and PR1C. The tree was constructed using neighbor-joining in MEGA 5.0 with 500 bootstrap replicates. Coefficients are indicated below the respective nodes. Domain architecture analyses were constructed with equal proportions of the respective sequences according to the results of Pfam databases.


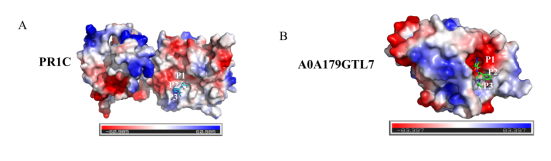


**Supplementary Figure 3.** Electrostatic potential distribution on the surface of PR1C and A0A179GTL7. The electrostatic potential is colored as a gradient from red (negative) to blue (positive). The peptide substrate's binding sites, which interact with protease binding pockets S4-S4', were indicated as P4, P3, P2, P1, P1', P2', P3', P4' in a sequence from N-terminal (N) to C-terminal (C). Cleavage of the substrate took place between P1 and P1'.

**Supplementary Table 1. Amino acid composition analysis of corn flour and yeast extract**

| **Amino Acid** | **Content (ESTD: mmol/L)** | |
| --- | --- | --- |
|  | **Corn flour** | **Yeast extract** |
| **Non-polar amino acid** |  |  |
| Ala | 65.5 | 50.6 |
| Val | 13.9 | 20.9 |
| Leu | 27.0 | 43.8 |
| Ile | 10.5 | 24.1 |
| Phe | 13.5 | 18.4 |
| Tyr | 8.6 | 6.8 |
| Pro | 47.0 | 21.8 |
| Gly | 138.0 | 40.2 |
| **Acidic/basic and polar amino acid** |  |  |
| Arg | 24.6 | 22.6 |
| Lys | 22.2 | 26.7 |
| His | 5.7 | 6.4 |
| Asp | 65.7 | 48.9 |
| Glu | 64.9 | 81.1 |
| **Neutral and polar amino acid** |  |  |
| Ser | 23.3 | 29.5 |
| Thr | 18.7 | 27.1 |
| Met | 5.3 | 12.2 |
| Cys | 3.9 | 2.8 |

**Supplementary Table 2. Primers used in this study**

| Primers | Sequence (5’-3’) |
| --- | --- |
| Actin-F | TCGTCGATGAAGATCAAGATCATC |
| Actin-R | CACATCTGCTGGAAGGTCGACAG |
| AreA-F | CATGGACCTTGATGCTCCTGGTG |
| AreA-R | ACATCATCGTACTCGAGATGGAG |
| SREP-F | CAGCGTCGATAAGGGAGAGTACAC |
| SREP-R | GTATCGTTCCCTTTGCTCATGTTC |
| Asd-4-F | TCAGTTGGAGGCAGTGCAAGAGAG |
| Asd-4-R | CATCGTCAAGTTCCTCAGCAAGATC |
| LreA-F | CACTCCCAGCTTGATCTTGGTGAC |
| LreA-R | CACCTTCTCCATCTGTCTCAACTC |
| A0A179HGL4-F | CCAGCTTCGCAGATGGTGAAATG |
| A0A179HGL4-R | AGGATGCAAGGTCAGTTGTCTTG |
| NsdD-F | ACGGATCGAGAAGTCTCGGAGATG |
| NsdD-R | ATGTCGTGGTCCTCTTCGTACGTG |
| A0A179GPS9-F | GTCAGCGATTCGATGAGCCAATC |
| A0A179GPS9-R | ACCCTCGAGGTTCTCCTGGTCAG |
| A0A179GJC0-F | CAATATCTGCGATTGCGAAGTTG |
| A0A179GJC0-R | GTTCCTGCGTTGATGGCTGTTAG |
